# Supplementary figures and images for: Transplantation of chicken egg white extract-induced rabbit PBMCs as a treatment for renal ischemia-reperfusion injury in rabbits
Source: PLoS One. 2020 Dec 28;15(12):e0244160. doi: 10.1371/journal.pone.0244160 (PMC7769466; doi:10.1371/journal.pone.0244160)

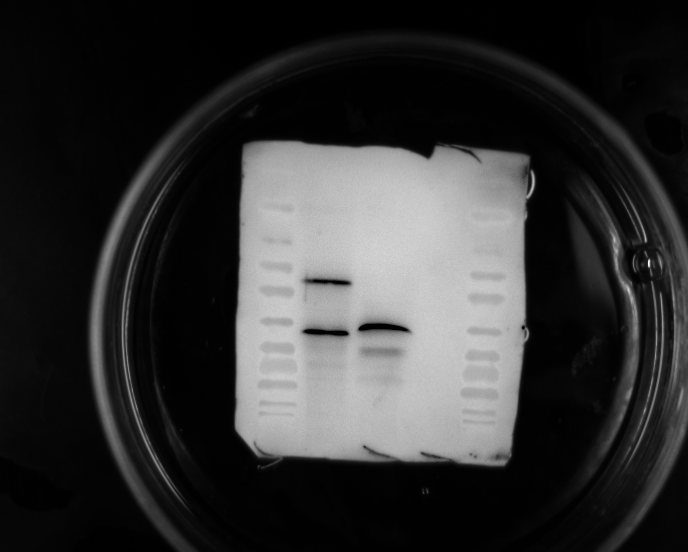

Supplement: S1 Fig — (TIF) [file pone.0244160.s001.tif]

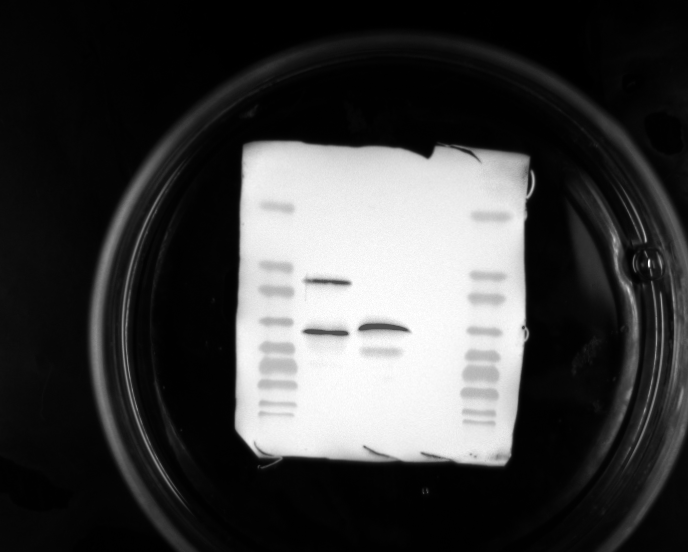

Supplement: S2 Fig — (TIF) [file pone.0244160.s002.tif]

## Slide 1
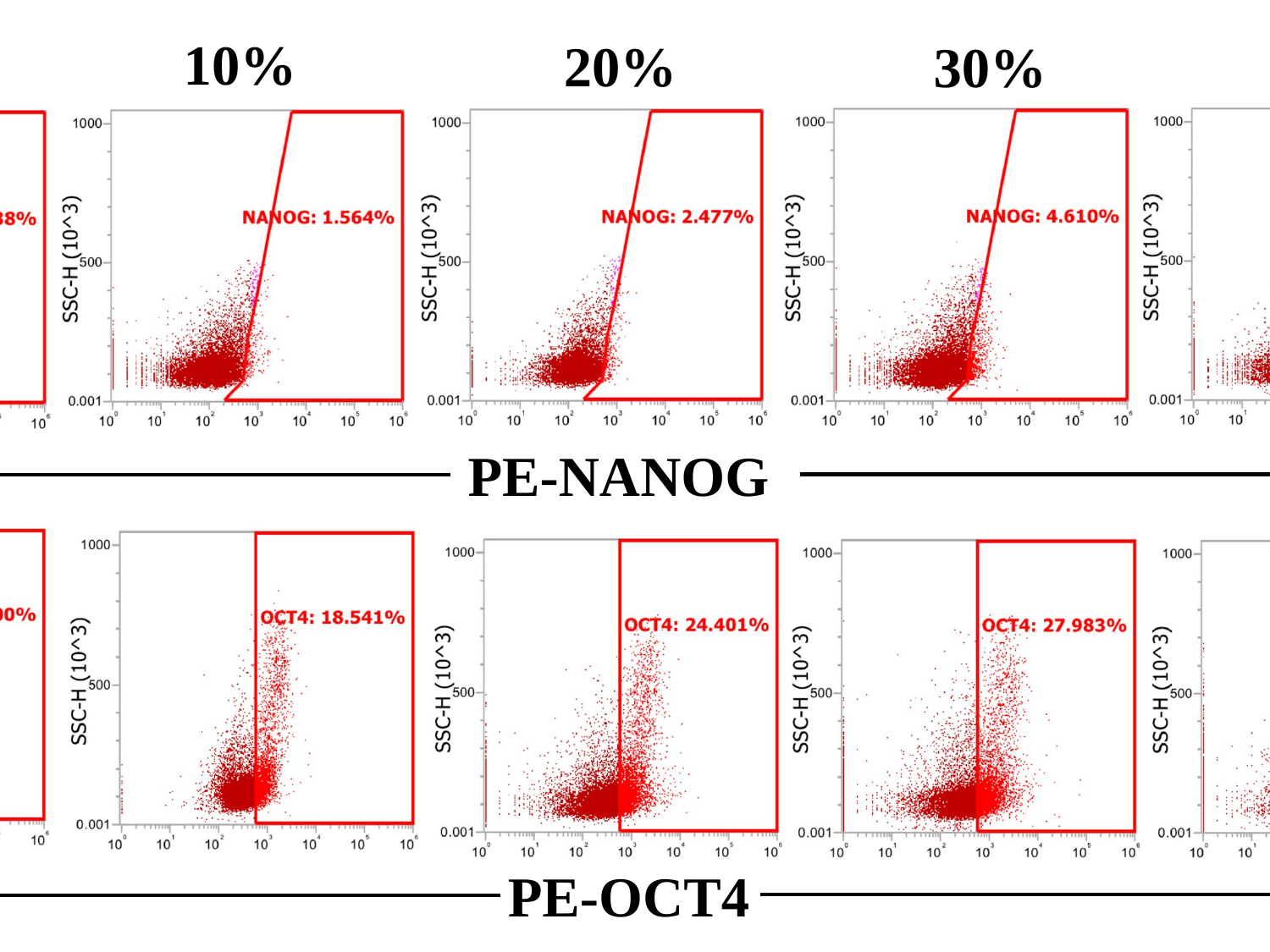

10%
Control
IgG
20%
30%
40%
50%
PE-NANOG
PE-OCT4

## Slide 2
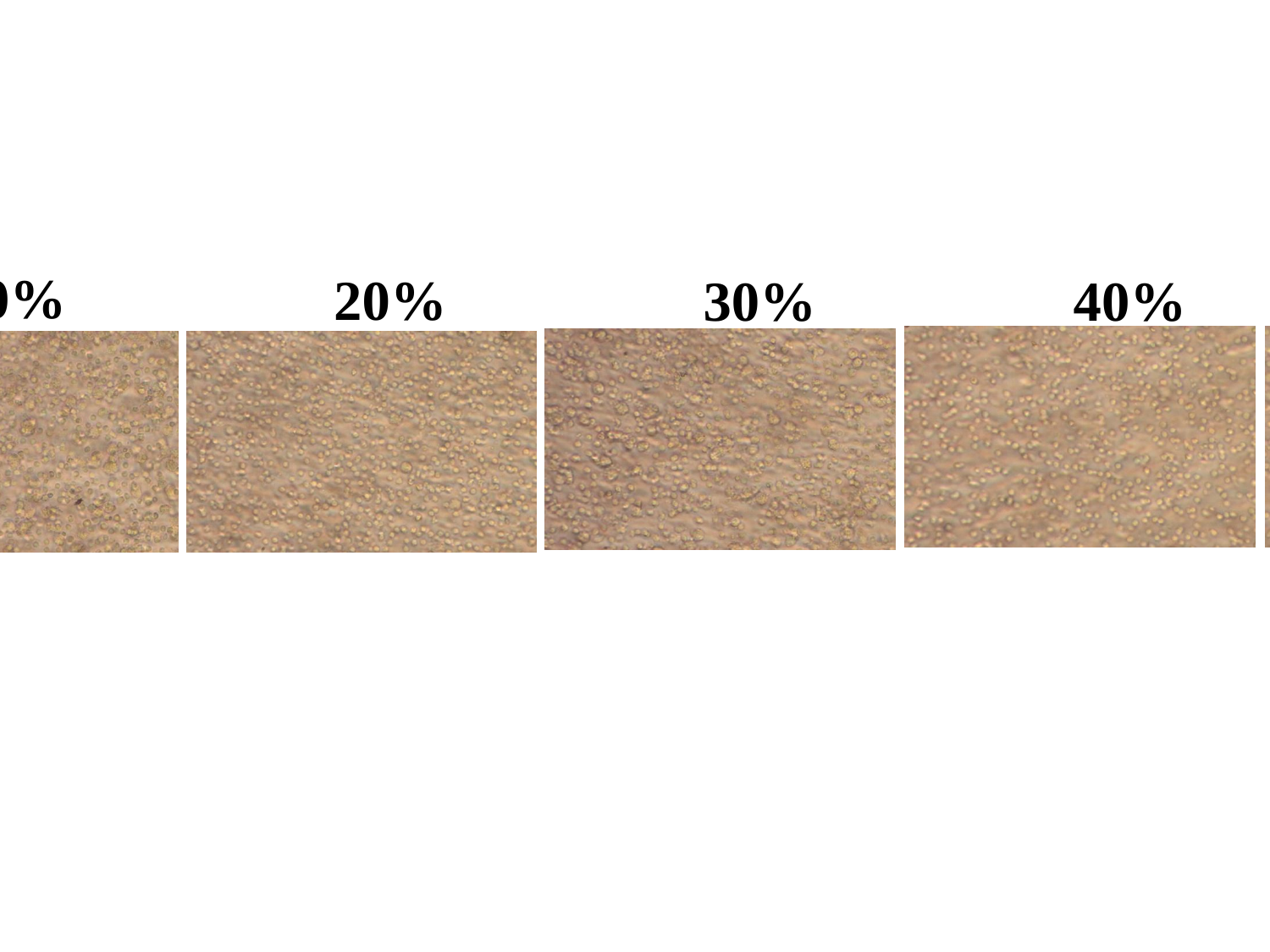

10%
Control
20%
30%
40%
50%

Supplement: S3 Fig — (PPTX) [file pone.0244160.s003.pptx]
