# Supplementary material for: Transplantation of chicken egg white extract-induced rabbit PBMCs as a treatment for renal ischemia-reperfusion injury in rabbits
Source: PLoS One. 2020 Dec 28;15(12):e0244160. doi: 10.1371/journal.pone.0244160 (PMC7769466; doi:10.1371/journal.pone.0244160)
Supplement: Attachment — Submitted filename: 2-Response to Reviewers.doc [file pone.0244160.s004.doc]

PONE-D-20-22977
Transplantation of Chicken Egg-White Extract-Induced Rabbit PBMCs as Treatment for Renal Ischemia-Reperfusion Injury in Rabbits
PLOS ONE

Dear Dr. Ruan,

Thank you for submitting your manuscript to PLOS ONE. After careful consideration, we feel that it has merit but does not fully meet PLOS ONE’s publication criteria as it currently stands. Therefore, we invite you to submit a revised version of the manuscript that addresses the points raised during the review process.

During revision, please give proper consideration to all the comments received from the reviewer. In addition, please try to remain consistent while discussing your results and do not exaggerate it. For example, in this manuscript you have not shown any similarity between your induced cells and mesenchymal stem cells. Hence, avoid any irrelevant discussion that does not support your findings. Furthermore, claiming the induced cells have dedifferentiated into pluripotent cells is a kind of exaggeration. Please try to make your remarks softer and realistic.     
We have revised the paper to make our remarks softer and more realistic. We changed “pluripotent cells” to “multipotent cells” to show that the cells we obtained are more mature.
Please submit your revised manuscript by 25 September 2020. If you will need more time than this to complete your revisions, please reply to this message or contact the journal office at [plosone@plos.org](mailto:plosone@plos.org). When you're ready to submit your revision, log on to https://www.editorialmanager.com/pone/ and select the 'Submissions Needing Revision' folder to locate your manuscript file.

Please include the following items when submitting your revised manuscript:

- A rebuttal letter that responds to each point raised by the academic editor and reviewer(s). You should upload this letter as a separate file labeled 'Response to Reviewers'.
- A marked-up copy of your manuscript that highlights changes made to the original version. You should upload this as a separate file labeled 'Revised Manuscript with Track Changes'.
- An unmarked version of your revised paper without tracked changes. You should upload this as a separate file labeled 'Manuscript'.

If you would like to make changes to your financial disclosure, please include your updated statement in your cover letter. Guidelines for resubmitting your figure files are available below the reviewer comments at the end of this letter.

If applicable, we recommend that you deposit your laboratory protocols in protocols.io to enhance the reproducibility of your results. Protocols.io assigns your protocol its own identifier (DOI) so that it can be cited independently in the future. For instructions see: [http://journals.plos.org/plosone/s/submission-guidelines#loc-laboratory-protocols](http://journals.plos.org/plosone/s/submission-guidelines#loc-laboratory-protocols)

We look forward to receiving your revised manuscript.

Kind regards,

Nazmul Haque
Academic Editor
PLOS ONE

Dear Editor,

Thank you very much for your letter and advice. We have revised the paper and would like to resubmit it for your consideration. We have addressed the reviewers’ comments. This manuscript has been edited by American Journal Experts.

We hope that our revised manuscript is acceptable for publication, and we look forward to hearing from you at your earliest convenience.

Best wishes,

Guang-ping Ruan

We would like to express our sincere thanks to the reviewers for their constructive and positive comments.

Journal Requirements:

When submitting your revision, we need you to address these additional requirements.

1. Please ensure that your manuscript meets PLOS ONE's style requirements, including those for file naming. The PLOS ONE style templates can be found at

https://journals.plos.org/plosone/s/file?id=wjVg/PLOSOne_formatting_sample_main_body.pdf and

https://journals.plos.org/plosone/s/file?id=ba62/PLOSOne_formatting_sample_title_authors_affiliations.pdf

 We have ensured that our manuscript meets PLOS ONE's style requirements, including those for file naming.

2. In your Methods section, please provide additional details regarding the rabbits used in your study and ensure you have described the source. For more information regarding PLOS' policy on materials sharing and reporting, see https://journals.plos.org/plosone/s/materials-and-software-sharing#loc-sharing-materials.

The rabbits were purchased from Kunming Chushang Technology Co., Ltd., and the license number was SCXK (Dian) K2018-0001.

3. In your Methods section, please state the volume of the blood samples collected for use in your study.

Twenty milliliters of peripheral blood was harvested from one rabbit. At the same time, the peripheral blood of 3 rabbits was collected, and the experiment was performed more than 3 times to ensure repeatability.

4. PLOS ONE now requires that authors provide the original uncropped and unadjusted images underlying all blot or gel results reported in a submission’s figures or Supporting Information files. This policy and the journal’s other requirements for blot/gel reporting and figure preparation are described in detail at https://journals.plos.org/plosone/s/figures#loc-blot-and-gel-reporting-requirements and https://journals.plos.org/plosone/s/figures#loc-preparing-figures-from-image-files. When you submit your revised manuscript, please ensure that your figures adhere fully to these guidelines and provide the original underlying images for all blot or gel data reported in your submission. See the following link for instructions on providing the original image data: https://journals.plos.org/plosone/s/figures#loc-original-images-for-blots-and-gels.

In your cover letter, please note whether your blot/gel image data are in Supporting Information or posted at a public data repository, provide the repository URL if relevant, and provide specific details as to which raw blot/gel images, if any, are not available. Email us at[plosone@plos.org](mailto:plosone@plos.org) if you have any questions.

 We have provided the original image in Supporting Information.

5. To comply with PLOS ONE submissions requirements, in your Methods section, please provide additional information on the animal research and ensure you have included details on efforts to alleviate animal suffering.

All experimental protocols were approved by the Experimental Animal Ethics Committee of the 920th Hospital of the Joint Logistics Support Force of the People's Liberation Army (approved number: 2019-002-01).

6. Thank you for stating the following in the Acknowledgments Section of your manuscript:

[This work was supported by grants from Yunnan Science and Technology Plan Project
Major Science and Technology Project (2018ZF007) and the Yunnan Province
Applied Basic Research Program Key Project (2018FA041, 2017FA040). National
Natural Science Foundation (31970515), 920th Hospital of the PLA Joint Logistics
Support Force In-hospital technology plans (2019YGB17, 2019YGA05).]

We note that you have provided funding information that is not currently declared in your Funding Statement. However, funding information should not appear in the Acknowledgments section or other areas of your manuscript. We will only publish funding information present in the Funding Statement section of the online submission form.

Please remove any funding-related text from the manuscript and let us know how you would like to update your Funding Statement. Currently, your Funding Statement reads as follows:

 [The funders had no role in study design, data collection and analysis, decision to publish, or preparation of the manuscript.]

 We have removed funding-related text from the manuscript and would not like to update our Funding Statement.

7. Your ethics statement must appear in the Methods section of your manuscript. If your ethics statement is written in any section besides the Methods, please move it to the Methods section and delete it from any other section. Please also ensure that your ethics statement is included in your manuscript, as the ethics section of your online submission will not be published alongside your manuscript.

Our ethics statement is included in the Methods section of our manuscript.

[Note: HTML markup is below. Please do not edit.]

Reviewers' comments:

Reviewer's Responses to Questions

**Comments to the Author**

1. Is the manuscript technically sound, and do the data support the conclusions?

The manuscript must describe a technically sound piece of scientific research with data that supports the conclusions. Experiments must have been conducted rigorously, with appropriate controls, replication, and sample sizes. The conclusions must be drawn appropriately based on the data presented.

Reviewer #1: Yes

Reviewer #2: Partly

Reviewer #3: No

2. Has the statistical analysis been performed appropriately and rigorously?

Reviewer #1: Yes

Reviewer #2: Yes

Reviewer #3: Yes

3. Have the authors made all data underlying the findings in their manuscript fully available?

The [PLOS Data policy](http://www.plosone.org/static/policies.action#sharing) requires authors to make all data underlying the findings described in their manuscript fully available without restriction, with rare exception (please refer to the Data Availability Statement in the manuscript PDF file). The data should be provided as part of the manuscript or its supporting information, or deposited to a public repository. For example, in addition to summary statistics, the data points behind means, medians and variance measures should be available. If there are restrictions on publicly sharing data—e.g. participant privacy or use of data from a third party—those must be specified.

Reviewer #1: Yes

Reviewer #2: No

Reviewer #3: No

4. Is the manuscript presented in an intelligible fashion and written in standard English?

PLOS ONE does not copyedit accepted manuscripts, so the language in submitted articles must be clear, correct, and unambiguous. Any typographical or grammatical errors should be corrected at revision, so please note any specific errors here.

Reviewer #1: Yes

Reviewer #2: Yes

Reviewer #3: No

5. Review Comments to the Author

Please use the space provided to explain your answers to the questions above. You may also include additional comments for the author, including concerns about dual publication, research ethics, or publication ethics. (Please upload your review as an attachment if it exceeds 20,000 characters)

Responses to Reviewer 1

Reviewer #1: The idea for this research is interesting and offers a numerous possibilities for future investigations such as clarification of strict molecular mechanism of beneficial induced PBMC treatment.
The basic question is how the authors decided to conduct just three procedures of induced PBMC administration. Why would not we suppose that perhaps one treatment is enough for restoration of kidney architecture and function?

When stem cells are used to treat kidney disease, they usually have to be administered 3 times, once a week for 3 consecutive weeks, so we also adopted this course of treatment. In the preliminary experiment, we also conducted one treatment, and the effect was not as good as that of three treatments.

Why did not you analyze animal blood sample after first week of treatment? Please explain and the answers on these question incorporate in discussion. Additionally, restructure some part of discussion by comparing your results with other studies instead of simple paraphrase of already shown results.

We also analyzed animal blood samples after the first week of treatment. However, the difference between the four groups was not significant, so in the results, we show only the results obtained for the 4 groups after 3 treatments. We have provided answers to these questions in the discussion.

We have restructured some parts of the discussion by comparing our results with our previous research instead of simply paraphrasing previously shown results. We have added the following sentences in the discussion: “We successfully established a rabbit model of renal interstitial fibrosis and demonstrated that transplantation of induced autologous stem cells can repair kidney damage within 8 weeks[24]. Stem cells provide an advantageous microenvironment for the repair of renal tubular epithelial damage[24]. In this experiment, after the administration of 3 consecutive weekly treatments, the serum creatinine and urea nitrogen levels in the induced cell treatment group were restored to the same level observed in the normal control group, whereas the levels in the noninduced cell treatment and model control groups were significantly elevated.”

Please, use uniform phrase ACUTE KIDNEY INJURY, not ACUTE RENAL FAILURE.

We have revised the paper and consistently used the phrase “acute kidney injury” rather than “acute renal failure”.

Responses to Reviewer 2

Reviewer #2: The current manuscript entitled ‘Transplantation of Chicken Egg-White Extract-Induced Rabbit PBMCs as Treatment for Renal Ischemia-Reperfusion Injury in Rabbits’ by RUAN et al. is moderately organised and represented. However the following points need to be addressed for betterment of their manuscript.

1# In the Materials and Methods section the author wrote that ‘As shown in our previous study, cells grown in 50% chicken egg-white extract culture medium will differentiate into pluripotent stem cells and may be used to treat disease.’ They need to cite their paper here.
We have cited our paper here: “As shown in our previous study, cells grown in 50% chicken egg-white extract in culture medium will differentiate into multipotent stem cells and may be used to treat diseases[9].”
2# To check the stemness property the author checked the expression level of Oct4, Nanog and Sox2. I wonder why they overlooked Klf4 and c-Myc detection. To characterize any induced pluripotent cell type OKSM expression is very important.
Although there are many markers of pluripotent stem cells, such as Oct4, Nanog, Sox2, Klf4 and c-Myc, the most representative and the most tested markers are Oct4, Nanog and Sox2. Therefore, we tested only Oct4, Nanog and Sox2.
3# It is highly recommended to draw a diagram of this research where all treatment groups will be included for better understanding.
Our experimental design includes 4 groups: the normal control group, model group, uninduced cell therapy group and induced cell therapy group. The efficacy comparison was carried out among the 4 groups. If all the treatment groups were included in the figure, it would not be possible to make a very good comparison. At present, our results show the comparison of 4 groups, which can better show the effect.
4# In the histopathological assessment, the author performed immunohistochemistry analysis of the fibrosis factor TGF-β only. Is it adequate to represent fibrosis by detecting only Tgf-b expression? The authors need to add couple of more markers.
In addition to performing immunohistochemistry analysis of the fibrosis-related factor TGF-β, we also carried out Masson’s trichrome staining and renal PAS staining. These experiments all represent pathological changes in the kidney.
5# In the result section, discussion under the heading of ‘Infusions of labeled PBMCs to treat the rabbit model’ is not sufficient. The author should write their findings very clearly in this section. It is highly recommended to summarize each result of every sub-category at the end of description.
We have revised the heading ‘Infusions of labeled PBMCs to treat the rabbit model’ to “Successful labeling of PBMCs with CFSE”. We rewrote our findings very clearly in this section as follows: “Carboxyfluorescein diacetate and succinimidyl ester (CFSE)-labeled PBMCs exhibited yellow-green fluorescence under a fluorescence microscope, as shown in Fig 1B. After counterstaining with DAPI, all cells showed blue fluorescence, as shown in Fig 1A, and when the fluorescence staining overlapped, the cells were successfully labeled, as shown in Fig 1C.”
6# In the result section, the author wrote ‘Statistical analysis showed that the two groups were statistically significant (n=3, p=0.003)’ regarding Figure 1D. However, I failed to find any indication of this significance in the figure!!! Furthermore, the author mentioned that the expression of the somatic cell gene LMNA was decreased. But Figure 1D does not show any difference among the two candidates.
We have added “*” to indicate this significance in Figure 1D. We also added the following sentence: “The difference in the somatic cell gene LMNA between the two groups was not statistically significant.”
7# For statistical analysis they mentioned n=3 in everywhere. Does it represent biological replicates or technical replicates? In case of quantitative PCR do they use biological replicates? Author need to mention it in their manuscript.
It represents biological replicates. For quantitative PCR, we used biological replicates: “We induced the cells 3 times and performed 3 biological replicates.”
8# Author needs to change all gene symbols in Italic throughout the manuscript including Tables also.
We have presented all gene symbols in italics throughout the manuscript, including in Tables.
9# The picture resolution of Figure 1E-1M is very poor. The numbers and description are very hard to read. Furthermore, authors used SSEA-4 candidate to show the status of pluripotency induction. But they did not show SSEA-4 transcript expression. It would be better if they synchronize their candidates for qPCR and Flow cytometric analysis.
We have recreated Figure 1E-1M, and now the numbers and descriptions are very easy to read. Since SSEA-4 is expressed on the cell surface and not in the nucleus, quantitative PCR detection cannot be performed, but flow cytometric detection can be performed.
10# In the methods section the author needs to add the name and company of instruments they used in this research. It is recommended to check the manuscript again and correct missing one.
In the methods section, we have added the names and manufacturers of instruments we used in this research: “Enhanced chemiluminescence (ECL) was used to detect the proteins, and images were captured using a chemiluminescence imaging camera (Tanon 5200, Shanghai).”
11# In the Figure 2, the labelling of A-D is missing. Additionally, which one is noninduced and induced PBMCs? Authors need to be more careful to organise a manuscript.
In Figure 2, the labeling of A-D is in the lower right corner of each picture. We organized the manuscript more carefully and added the following sentences: “Fig 2A and 2C show uninduced PBMCs, and Fig 2B and 2D show induced PBMCs. Fig 2A and 2B show OCT4 expression, and Fig 2C and 2D show NANOG expression.”

12# In the Figure 2E, it is recommended to replace 1-5 numbering by adding text. And also mention candidates as usual.
In Figure 2E, we have replaced the numbers 1-5 with added text. Additionally, candidates are mentioned.
13# In Figure 2F, the picture resolution is very poor. Failed to read the figure text. There is no sign of statistically significance though author claim it in the manuscript.
In Figure 2F,	the picture resolution is now acceptable, and the figure text can be read. There is also an indication of statistical significance as reported in the manuscript.
14# In the Figure 2G-2I, I failed to read the text of it. Most probably, the statistical significance signs are also missing here.
Since Figure 2 contains many pictures, the text is not very clear, but if you open the figure in illustration software, the text is clear. In Figure 2G-2I, the indicators of statistical significance are also presented.
15# The Figure 3A-3D is difficult to understand. The difference is not visible. Author needs to reedit their picture for better visualisation. Furthermore, 3A-3H figures have no identifying text!!!
In Figure 3A-3D, if you open the image in illustration software, you can see the obvious difference. We have added the identifying text in Figures 3A-3H.
16# The Figure 3I-3J is difficult to read. Most probably, the statistical significance signs are also missing here!!!
In Figure 3I, * indicates P<0.05 compared to the induced group. In Figure 3J, * indicates P<0.05 compared to the model group.
17# In the Figure 4, authors are asked to label A-H, to add statistical significance sign in I and to add text for magnification bar in J. Furthermore, the author wrote ‘Immunofluorescence results showed that the transplanted cells had green fluorescence and red fluorescence at the same time, indicating that the transplanted cells were differentiated into tubular epithelial cells.’ But it is very hard to find any red fluorescence in Figure 4J. Need to use arrow for any specific presentation.
In Figure 4, we have labeled A-H and added an indicator of statistical significance in I. * indicates P<0.05 compared to the model group. The magnification bar in J is 100 µm. We have used an arrow to indicate red fluorescence in Figure 4J.
18# In Figure 5, the author needs to add line bars among their comparable candidates in the figure. It is hardly detectable among which the statistical analysis was done.
In Figure 5, we have added lines between the comparable candidates in the figure.
19# In Figure 6, the author needs to edit all texts. Nothing is visible here. It is very tough to comment more than that….!!!
In Figure 6, the darker the bubble color and the larger the volume are, the more significant the difference. In Figures 6A and 6B, we can see that the difference in the pyrimidine metabolism pathway is significant, as shown by the arrows. In Figures 6C and 6D, we can see that the differences in phenylalanine, tyrosine and tryptophan biosynthesis pathways are significant, as indicated by the arrows.
20# The author wrote in summary ‘the results of this study suggest that the intravenous transplantation of induced PBMCs promotes the repair of acute kidney injury’. However, they did not check any expression of marker genes. They need to add couple of marker gene expressions, if possible.
As shown in Figure 3A-3D, the distribution of fluorescently labeled induced PBMCs in frozen kidney sections suggested that these cells were involved in repairing the injured kidney. The kidney tissues of the treatment group were frozen sectioned, incubated with a primary antibody for aquaporin-1 (from Abcam, ab9566, Cambridge, UK) that stains the proximal tubules, incubated with a red fluorescence-labeled secondary antibody (from Abcam, ab150115, Cambridge, UK), and counterstained with DAPI, and the distribution of red, green, and blue fluorescence was observed under a fluorescence microscope. Immunofluorescence results showed that the transplanted cells exhibited green fluorescence and red fluorescence at the same time, indicating that the transplanted cells were differentiated into tubular epithelial cells (TECs) (Figure 4J). We detected the expression of the aquaporin-1 protein, which is expressed during the development of the renal tubules, and the results showed that CFSE-labeled PBMCs were transformed into renal tubules.
21# Authors need to submit their metabolomics data in any open access platform.

Yes, we will submit our metabolomics data to an open access platform.

Responses to Reviewer 3

Reviewer #3: The manuscript, “Transplantation of Chicken Egg-White Extract-Induced Rabbit PBMCs as Treatment for Renal Ischemia-Reperfusion Injury in Rabbits” does not present any novel scientific concept. The study was not presented properly, the figures were not labelled and there are number of unclear images presented without paying due attention.
• Induction of PBMCs by chicken egg white extract is an old concept. Number of studies have been conducted two-three decades ago. Heterogeneous nature of the chicken egg white components and allergenic substances could affect the outcome.
This is the first report to use chicken egg-white extract to induce PBMCs and treat rabbit renal ischemia-reperfusion injury. The induction effect of the chicken egg-white extract obtained with our method was stable.

• Authors need to explain the results of the previous studies with egg white extract on inducing cells in the introduction.
We have added a sentence in the introduction: “In our previous research, we found that chicken egg white extract can induce the reverse differentiation of somatic cells into multipotent stem cells[5, 9].”

• Chicken egg white contains different growth factors and enzymes. Which component of chicken egg white are you interested in and expecting to induce cells?
After our experimental research, it was found that a protein in the chicken egg-white extract was the active factor.

• The research gap addressed by the study is not well defined.
We have added a sentence in the conclusion: “Future research needs to identify the key molecules in chicken egg-white extract to further improve the induction efficiency.”

• How many rabbits were used for harvesting peripheral blood? Please explain the procedure and mention how the animal well-being was maintained.
Twenty milliliters of peripheral blood was harvested from one rabbit. At the same time, the peripheral blood of 3 rabbits was collected, and the experiment was performed more than 3 times to ensure repeatability. All experimental protocols were approved by the Experimental Animal Ethics Committee of the 920th Hospital of the Joint Logistics Support Force of the People's Liberation Army (approved number: 2019-002-01).

• What is the rationale of using 50% egg white extract medium? How was it formulated? Was the protocol standardized? If so, please cite the methodology.
Our previous experiments show that with 50% chicken egg-white extract, cell growth is not affected, and the cell induction efficiency is high. Refer to references 5 and 9.

• Instead of formulating egg white extract medium, authors could have used purified egg white lysozymes as done in other studies to avoid interferences with host biology.
Since the antibodies we use are all anti-rabbit antibodies, and the source of the chicken egg-white extract is chickens, host factors will not interfere with our flow cytometry and WB experiments.

• Methodology is not sub-sectioned well. Please put a title instead of using sentences and explain the methodology thereafter.
We have added a title instead of using sentences and explained the methodology thereafter.

• Results section also needs proper headings rather than using sentences
We have revised the results section using proper headings rather than using sentences.

• In the discussion, the results of the egg white extract induction mechanism were not discussed.
We have added sentences in the discussion, and the results related to the induction mechanism of egg-white extract were discussed, as follows: “Studies have shown that mammalian egg cell and Xenopus egg cell extracts can reprogram somatic cells[6, 7]. Our previous research has shown that chicken egg extract can also reprogram somatic cells[5, 9]. The chicken egg is the largest egg cell; thus, its ability to reprogram somatic cells will advance cell biological research.”

• Please mention other methods that have been used for induction, and compare the results of those methods with egg white extract method.
We have compared the results of other methods with the egg-white extract method in the discussion: “Mammalian egg cell and Xenopus egg cell extract-induced reprogramming steps are cumbersome, and the extracts are difficult to obtain, but chicken eggs are the largest egg cells, and a large amount of chicken egg white extract can be obtained. If the procedure is performed in a sterile manner, the obtained extract does not need to be filtered and sterilized. The activity of the extract can be maintained well.”

• Figures are not labelled. Therefore, referring to the figures is very difficult.
We have revised all the figures, and the figures are now labelled.

• The axes of the graphs are not labelled properly.
We have labeled the axes of the graphs properly.

• There are number of unclear images presented without paying due attention.
The picture is not clear because the large image is composed of many small pictures. The figure will be clearer if you open the picture with illustration software.

• The manuscript needs revision for English language.

We have submitted our paper to AJE for further revision.

6. PLOS authors have the option to publish the peer review history of their article ([what does this mean?](https://journals.plos.org/plosone/s/editorial-and-peer-review-process#loc-peer-review-history)). If published, this will include your full peer review and any attached files.


If you choose “no”, your identity will remain anonymous but your review may still be made public.


**Do you want your identity to be public for this peer review?** For information about this choice, including consent withdrawal, please see our [Privacy Policy](https://www.plos.org/privacy-policy).

Reviewer #1: No

Reviewer #2: No

Reviewer #3: No

[NOTE: If reviewer comments were submitted as an attachment file, they will be attached to this email and accessible via the submission site. Please log into your account, locate the manuscript record, and check for the action link "View Attachments". If this link does not appear, there are no attachment files.]

While revising your submission, please upload your figure files to the Preflight Analysis and Conversion Engine (PACE) digital diagnostic tool, <https://pacev2.apexcovantage.com/>. PACE helps ensure that figures meet PLOS requirements. To use PACE, you must first register as a user. Registration is free. Then, login and navigate to the UPLOAD tab, where you will find detailed instructions on how to use the tool. If you encounter any issues or have any questions when using PACE, please email PLOS at [figures@plos.org](mailto:figures@plos.org). Please note that Supporting Information files do not need this step.

*In compliance with data protection regulations, you may request that we remove your personal registration details at any time.* [*(Remove my information/details)*](https://www.editorialmanager.com/pone/login.asp?a=r)*. Please contact the publication office if you have any questions.*
