# Supplementary material for: Transplantation of chicken egg white extract-induced rabbit PBMCs as a treatment for renal ischemia-reperfusion injury in rabbits
Source: PLoS One. 2020 Dec 28;15(12):e0244160. doi: 10.1371/journal.pone.0244160 (PMC7769466; doi:10.1371/journal.pone.0244160)
Supplement: Attachment — Submitted filename: 3-Response to Reviewers.doc [file pone.0244160.s005.doc]

PONE-D-20-22977R1
Transplantation of Chicken Egg-White Extract-Induced Rabbit PBMCs as a Treatment for Renal Ischemia-Reperfusion Injury in Rabbits
PLOS ONE

Dear Dr. Ruan,

Thank you for submitting your manuscript to PLOS ONE. After careful consideration, we feel that it has merit but does not fully meet PLOS ONE’s publication criteria as it currently stands. Therefore, we invite you to submit a revised version of the manuscript that addresses the points raised during the review process.

Please submit your revised manuscript by 06 November 2020. If you will need more time than this to complete your revisions, please reply to this message or contact the journal office at [plosone@plos.org](mailto:plosone@plos.org). When you're ready to submit your revision, log on to https://www.editorialmanager.com/pone/ and select the 'Submissions Needing Revision' folder to locate your manuscript file.

Please include the following items when submitting your revised manuscript:

- A rebuttal letter that responds to each point raised by the academic editor and reviewer(s). You should upload this letter as a separate file labeled 'Response to Reviewers'.
- A marked-up copy of your manuscript that highlights changes made to the original version. You should upload this as a separate file labeled 'Revised Manuscript with Track Changes'.
- An unmarked version of your revised paper without tracked changes. You should upload this as a separate file labeled 'Manuscript'.

If you would like to make changes to your financial disclosure, please include your updated statement in your cover letter. Guidelines for resubmitting your figure files are available below the reviewer comments at the end of this letter.

If applicable, we recommend that you deposit your laboratory protocols in protocols.io to enhance the reproducibility of your results. Protocols.io assigns your protocol its own identifier (DOI) so that it can be cited independently in the future. For instructions see: [http://journals.plos.org/plosone/s/submission-guidelines#loc-laboratory-protocols](http://journals.plos.org/plosone/s/submission-guidelines#loc-laboratory-protocols)

We look forward to receiving your revised manuscript.

Kind regards,

Nazmul Haque
Academic Editor
PLOS ONE

Dear Editor,

Thank you very much for your letter and advice. We have revised the paper and would like to resubmit it for your consideration. We have addressed the reviewers’ comments. This manuscript has been edited by American Journal Experts.

We hope that our revised manuscript is acceptable for publication, and we look forward to hearing from you at your earliest convenience.

Best wishes,

Guang-ping Ruan

We would like to express our sincere thanks to the reviewers for their constructive and positive comments.

[Note: HTML markup is below. Please do not edit.]

Reviewers' comments:

Reviewer's Responses to Questions

**Comments to the Author**

1. If the authors have adequately addressed your comments raised in a previous round of review and you feel that this manuscript is now acceptable for publication, you may indicate that here to bypass the “Comments to the Author” section, enter your conflict of interest statement in the “Confidential to Editor” section, and submit your "Accept" recommendation.

Reviewer #1: All comments have been addressed

Reviewer #3: (No Response)

2. Is the manuscript technically sound, and do the data support the conclusions?

The manuscript must describe a technically sound piece of scientific research with data that supports the conclusions. Experiments must have been conducted rigorously, with appropriate controls, replication, and sample sizes. The conclusions must be drawn appropriately based on the data presented.

Reviewer #1: Yes

Reviewer #3: No

3. Has the statistical analysis been performed appropriately and rigorously?

Reviewer #1: Yes

Reviewer #3: Yes

4. Have the authors made all data underlying the findings in their manuscript fully available?

The [PLOS Data policy](http://www.plosone.org/static/policies.action#sharing) requires authors to make all data underlying the findings described in their manuscript fully available without restriction, with rare exception (please refer to the Data Availability Statement in the manuscript PDF file). The data should be provided as part of the manuscript or its supporting information, or deposited to a public repository. For example, in addition to summary statistics, the data points behind means, medians and variance measures should be available. If there are restrictions on publicly sharing data—e.g. participant privacy or use of data from a third party—those must be specified.

Reviewer #1: Yes

Reviewer #3: Yes

5. Is the manuscript presented in an intelligible fashion and written in standard English?

PLOS ONE does not copyedit accepted manuscripts, so the language in submitted articles must be clear, correct, and unambiguous. Any typographical or grammatical errors should be corrected at revision, so please note any specific errors here.

Reviewer #1: Yes

Reviewer #3: No

6. Review Comments to the Author

Please use the space provided to explain your answers to the questions above. You may also include additional comments for the author, including concerns about dual publication, research ethics, or publication ethics. (Please upload your review as an attachment if it exceeds 20,000 characters)

Reviewer #1: (No Response)

Responses to Reviewer 3

Reviewer #3: Although authors have revised the manuscript up to a certain degree, there are inadequately addressed concerns as mentioned below.

1. As a response to the reviewer's comments, authors have mentioned that the induction effect of the chicken egg white extract obtained according to their methodology is "stable", with our giving any supportive evidence. How do the authors justify this?
We repeated the induction experiment and performed flow cytometry, and the results showed that our induction experiment was stable. The result is as follows.

As the concentration of the egg white extract increases, the positive rate gradually increases, and the positive rate is the highest at a final concentration of 50%.
2. Authors have added only a single sentence on chicken egg white extract as the previous literature. There should be a more extensive literature review in the introduction in relation to this.
We have made a more extensive literature review in the introduction in relation to this. “In our laboratory, induced multipotent stem cells are generated by treating peripheral blood mononuclear cells (PBMCs) from rabbit peripheral blood with a homemade egg-white extract to reverse differentiation. This is the first report to use chicken egg-white extract to induce PBMCs and treat rabbit renal ischemia-reperfusion injury. Studies have shown that extracts of mammalian oocytes and Xenopus oocytes have the potential to reprogram cells. The identification of egg extracts with the ability to maintain and enhance the survival and differentiation of cells will be widely useful in cellular biology research. Many studies have reported that animal egg extracts are able to induce the reprogramming of somatic cells. The chicken egg yolk is the largest egg cell, where the yolk membrane comprises the cell membrane, and the egg white and eggshell, which have nutritional and protective roles, are formed by oviduct secretions. Therefore, chicken egg white extract has the capacity to induce stemness in PBMCs.”
3. What is the protein in chicken egg white extract that you consider as the active factor?
Because we have done the following experiments, using protease, DNase, and RNase to respectively lyse the protein, DNA, and RNA in the egg white extract, and then conduct our induction experiment, and found that the egg white extract after protein lysis no longer has the ability to reprogram cells, and the chicken protein extract after DNA and RNA lysis still has the ability to reprogram cells. So we proved that the main role of the extract is the protein component.
4. Research gap needs to be logically stated in the introduction.
We have added a sentence in the introduction: “Further experiments need to find the key molecules in the chicken egg white extract in order to further improve the induction efficiency and promote this method.”
5. How was the protocol on 50% egg white extract medium standardised?

“We repeated the induction experiment, using 10%, 20%, 30%, 40%, 50% final concentration of chicken egg-white extract to induce cells. As the concentration of the chicken egg-white extract increased, the pluripotency factor positive rate gradually increased, and at the final concentration of 50% the positive rate is the highest. But if the concentration of chicken egg-white extract exceeds 50%, cell growth will be affected. Thus, we used 50% chicken egg-white extract-induced rabbit PBMCs as a treatment for kidney injury in the rabbit model.” The result is shown below.

7. PLOS authors have the option to publish the peer review history of their article ([what does this mean?](https://journals.plos.org/plosone/s/editorial-and-peer-review-process#loc-peer-review-history)). If published, this will include your full peer review and any attached files.


If you choose “no”, your identity will remain anonymous but your review may still be made public.


**Do you want your identity to be public for this peer review?** For information about this choice, including consent withdrawal, please see our [Privacy Policy](https://www.plos.org/privacy-policy).

Reviewer #1: No

Reviewer #3: No

[NOTE: If reviewer comments were submitted as an attachment file, they will be attached to this email and accessible via the submission site. Please log into your account, locate the manuscript record, and check for the action link "View Attachments". If this link does not appear, there are no attachment files.]

While revising your submission, please upload your figure files to the Preflight Analysis and Conversion Engine (PACE) digital diagnostic tool, <https://pacev2.apexcovantage.com/>. PACE helps ensure that figures meet PLOS requirements. To use PACE, you must first register as a user. Registration is free. Then, login and navigate to the UPLOAD tab, where you will find detailed instructions on how to use the tool. If you encounter any issues or have any questions when using PACE, please email PLOS at [figures@plos.org](mailto:figures@plos.org). Please note that Supporting Information files do not need this step.

*In compliance with data protection regulations, you may request that we remove your personal registration details at any time.* [*(Remove my information/details)*](https://www.editorialmanager.com/pone/login.asp?a=r)*. Please contact the publication office if you have any questions.*
