# Supplementary material for: Transplantation of chicken egg white extract-induced rabbit PBMCs as a treatment for renal ischemia-reperfusion injury in rabbits
Source: PLoS One. 2020 Dec 28;15(12):e0244160. doi: 10.1371/journal.pone.0244160 (PMC7769466; doi:10.1371/journal.pone.0244160)
Supplement: Attachment — Submitted filename: 4-Response to Reviewers.doc [file pone.0244160.s006.doc]

PONE-D-20-22977R2
Transplantation of Chicken Egg-White Extract-Induced Rabbit PBMCs as a Treatment for Renal Ischemia-Reperfusion Injury in Rabbits
PLOS ONE

Dear Dr. Ruan,

Thank you for submitting your manuscript to PLOS ONE. After careful consideration, we feel that it has merit but does not fully meet PLOS ONE’s publication criteria as it currently stands. Therefore, we invite you to submit a revised version of the manuscript that addresses the points raised during the review process.

Please provide proper consideration to the Editor's comments and revise the manuscript carefully.

Please submit your revised manuscript by 23rd November 2020. If you will need more time than this to complete your revisions, please reply to this message or contact the journal office at [plosone@plos.org](mailto:plosone@plos.org). When you're ready to submit your revision, log on to https://www.editorialmanager.com/pone/ and select the 'Submissions Needing Revision' folder to locate your manuscript file.

Please include the following items when submitting your revised manuscript:

- A rebuttal letter that responds to each point raised by the academic editor and reviewer(s). You should upload this letter as a separate file labeled 'Response to Reviewers'.
- A marked-up copy of your manuscript that highlights changes made to the original version. You should upload this as a separate file labeled 'Revised Manuscript with Track Changes'.
- An unmarked version of your revised paper without tracked changes. You should upload this as a separate file labeled 'Manuscript'.

If you would like to make changes to your financial disclosure, please include your updated statement in your cover letter. Guidelines for resubmitting your figure files are available below the reviewer comments at the end of this letter.

If applicable, we recommend that you deposit your laboratory protocols in protocols.io to enhance the reproducibility of your results. Protocols.io assigns your protocol its own identifier (DOI) so that it can be cited independently in the future. For instructions see: [http://journals.plos.org/plosone/s/submission-guidelines#loc-laboratory-protocols](http://journals.plos.org/plosone/s/submission-guidelines#loc-laboratory-protocols)

We look forward to receiving your revised manuscript.

Kind regards,

Nazmul Haque
Academic Editor
PLOS ONE

Dear Editor,

Thank you very much for your letter and advice. We have revised the paper and would like to resubmit it for your consideration. We have addressed the reviewers’ comments. This manuscript has been edited by American Journal Experts.

We hope that our revised manuscript is acceptable for publication, and we look forward to hearing from you at your earliest convenience.

Best wishes,

Guang-ping Ruan

We would like to express our sincere thanks to the reviewers for their constructive and positive comments.

Additional Editor Comments (if provided):

1. Please remove the following sentences from the Materials and Methods and add in the discussion appropriately with proper citation. Mention the name of the cell type on which the experiment was conducted.
“We repeated the induction experiment, using 10%, 20%, 30%, 40%, 50% final concentration of chicken egg-white extract to induce cells. As the concentration of the chicken egg-white extract increased, the pluripotency factor positive rate gradually increased, and at the final concentration of 50% the positive rate is the highest. But if the concentration of chicken egg-white extract exceeds 50%, cell growth will be affected.”
We have removed the following sentences from the Materials and Methods and add in the discussion appropriately with proper citation. The name of the cell type on which the experiment was conducted was PBMCs. “We repeated the induction experiment, using 10%, 20%, 30%, 40%, 50% final concentration of chicken egg-white extract to induce PBMCs (see supplementary material). As the concentration of the chicken egg-white extract increased, the pluripotency factor positive rate gradually increased, and at the final concentration of 50% the positive rate is the highest. But if the concentration of chicken egg-white extract exceeds 50%, cell growth will be affected.”
2. In answer to the question 3 you have written the following sentences:
"Because we have done the following experiments, using protease, DNase, and RNase to respectively lyse the protein, DNA, and RNA in the egg white extract, and then conduct our induction experiment, and found that the egg white extract after protein lysis no longer has the ability to reprogram cells, and the chicken protein extract after DNA and RNA lysis still has the ability to reprogram cells. So we proved that the main role of the extract is the protein component.”

Please add these sentences in the discussion appropriately with proper citation.
We have added these sentences in the discussion appropriately. “Because we have done the following experiments, using protease, DNase, and RNase to respectively lyse the protein, DNA, and RNA in the egg white extract, and then conduct our induction experiment, and found that the egg white extract after protein lysis no longer has the ability to reprogram cells, and the chicken protein extract after DNA and RNA lysis still has the ability to reprogram cells. So we proved that the main role of the extract is the protein component (results are not displayed).”

[Note: HTML markup is below. Please do not edit.]


[NOTE: If reviewer comments were submitted as an attachment file, they will be attached to this email and accessible via the submission site. Please log into your account, locate the manuscript record, and check for the action link "View Attachments". If this link does not appear, there are no attachment files.]

While revising your submission, please upload your figure files to the Preflight Analysis and Conversion Engine (PACE) digital diagnostic tool, <https://pacev2.apexcovantage.com/>. PACE helps ensure that figures meet PLOS requirements. To use PACE, you must first register as a user. Registration is free. Then, login and navigate to the UPLOAD tab, where you will find detailed instructions on how to use the tool. If you encounter any issues or have any questions when using PACE, please email PLOS at [figures@plos.org](mailto:figures@plos.org). Please note that Supporting Information files do not need this step.

*In compliance with data protection regulations, you may request that we remove your personal registration details at any time.* [*(Remove my information/details)*](https://www.editorialmanager.com/pone/login.asp?a=r)*. Please contact the publication office if you have any questions.*
