# Supplementary material for: Transplantation of chicken egg white extract-induced rabbit PBMCs as a treatment for renal ischemia-reperfusion injury in rabbits
Source: PLoS One. 2020 Dec 28;15(12):e0244160. doi: 10.1371/journal.pone.0244160 (PMC7769466; doi:10.1371/journal.pone.0244160)
Supplement: Attachment — Submitted filename: 6-Response to Reviewers.doc [file pone.0244160.s007.doc]

PONE-D-20-22977R3
Transplantation of Chicken Egg-White Extract-Induced Rabbit PBMCs as a Treatment for Renal Ischemia-Reperfusion Injury in Rabbits
PLOS ONE

Dear Dr. Ruan,

Thank you for submitting your manuscript to PLOS ONE. After careful consideration, we feel that it has merit but does not fully meet PLOS ONE’s publication criteria as it currently stands. Therefore, we invite you to submit a revised version of the manuscript that addresses the points raised during the review process.

I appreciate your effort in addressing most of the issues raised by the reviewers. However, during this revision I would like to request you to provide proper consideration to the reviewers comments. Specially

1. Please revise the subtitles in the 'results' section and convert them into statements.

We have revised the titles of the subsections in the 'Results' section and converted them into statements.

2. Please revise the figures too, to make them more communicative.

We have revised the figures to better convey the information.

3. Provide proper explanation regarding data deposition.

We have deposited the data in protocols.io to enhance the reproducibility of our results. The DOI link is [dx.doi.org/10.17504/protocols.io.bpyrmpv6](https://dx.doi.org/10.17504/protocols.io.bpyrmpv6).

Please submit your revised manuscript by 13 December 2020. If you will need more time than this to complete your revisions, please reply to this message or contact the journal office at [plosone@plos.org](mailto:plosone@plos.org). When you're ready to submit your revision, log on to https://www.editorialmanager.com/pone/ and select the 'Submissions Needing Revision' folder to locate your manuscript file.

Please include the following items when submitting your revised manuscript:

- A rebuttal letter that responds to each point raised by the academic editor and reviewer(s). You should upload this letter as a separate file labeled 'Response to Reviewers'.
- A marked-up copy of your manuscript that highlights changes made to the original version. You should upload this as a separate file labeled 'Revised Manuscript with Track Changes'.
- An unmarked version of your revised paper without tracked changes. You should upload this as a separate file labeled 'Manuscript'.

If you would like to make changes to your financial disclosure, please include your updated statement in your cover letter. Guidelines for resubmitting your figure files are available below the reviewer comments at the end of this letter.
We have uploaded our figure files to the Preflight Analysis and Conversion Engine (PACE) digital diagnostic tool and ensured that the figures meet the PLOS requirements.
If applicable, we recommend that you deposit your laboratory protocols in protocols.io to enhance the reproducibility of your results. Protocols.io assigns your protocol its own identifier (DOI) so that it can be cited independently in the future. For instructions see: [http://journals.plos.org/plosone/s/submission-guidelines#loc-laboratory-protocols](http://journals.plos.org/plosone/s/submission-guidelines#loc-laboratory-protocols)
We have deposited the data in protocols.io to enhance the reproducibility of our results. The DOI link is [dx.doi.org/10.17504/protocols.io.bpyrmpv6](https://dx.doi.org/10.17504/protocols.io.bpyrmpv6).

We look forward to receiving your revised manuscript.

Kind regards,

Nazmul Haque
Academic Editor
PLOS ONE

Dear Editor,

Thank you very much for your letter and advice. We have revised the paper and would like to resubmit it for your consideration. We have addressed the reviewers’ comments, and the manuscript has been edited by American Journal Experts.

We hope that our revised manuscript is acceptable for publication and look forward to hearing from you at your earliest convenience.

Best wishes,

Guang-ping Ruan

We would like to express our sincere appreciation to the reviewers for their constructive and positive comments.

[Note: HTML markup is below. Please do not edit.]

Reviewers' comments:

Reviewer's Responses to Questions

**Comments to the Author**

1. If the authors have adequately addressed your comments raised in a previous round of review and you feel that this manuscript is now acceptable for publication, you may indicate that here to bypass the “Comments to the Author” section, enter your conflict of interest statement in the “Confidential to Editor” section, and submit your "Accept" recommendation.

Reviewer #2: (No Response)

Reviewer #3: All comments have been addressed

2. Is the manuscript technically sound, and do the data support the conclusions?

The manuscript must describe a technically sound piece of scientific research with data that supports the conclusions. Experiments must have been conducted rigorously, with appropriate controls, replication, and sample sizes. The conclusions must be drawn appropriately based on the data presented.

Reviewer #2: Partly

Reviewer #3: Yes

3. Has the statistical analysis been performed appropriately and rigorously?

Reviewer #2: Yes

Reviewer #3: Yes

4. Have the authors made all data underlying the findings in their manuscript fully available?

The [PLOS Data policy](http://www.plosone.org/static/policies.action#sharing) requires authors to make all data underlying the findings described in their manuscript fully available without restriction, with rare exception (please refer to the Data Availability Statement in the manuscript PDF file). The data should be provided as part of the manuscript or its supporting information, or deposited to a public repository. For example, in addition to summary statistics, the data points behind means, medians and variance measures should be available. If there are restrictions on publicly sharing data—e.g. participant privacy or use of data from a third party—those must be specified.

Reviewer #2: No

Reviewer #3: (No Response)

5. Is the manuscript presented in an intelligible fashion and written in standard English?

PLOS ONE does not copyedit accepted manuscripts, so the language in submitted articles must be clear, correct, and unambiguous. Any typographical or grammatical errors should be corrected at revision, so please note any specific errors here.

Reviewer #2: No

Reviewer #3: (No Response)

The manuscript has been edited by American Journal Experts again.

6. Review Comments to the Author

Please use the space provided to explain your answers to the questions above. You may also include additional comments for the author, including concerns about dual publication, research ethics, or publication ethics. (Please upload your review as an attachment if it exceeds 20,000 characters)

Responses to Reviewer 2

Reviewer #2: I would like to thank the editor for providing another opportunity to review the revised version of this current manuscript. I expected that there would be a significant change in the revised manuscript. However, it was not so. The following points are major issues to me –

The weakest part of this manuscript is the arrangement of their results. In the result section, they used 11 sub-headings to represent their results. Do any of the sub-headings represent a result? They used the experiment names only. Interpretation of result shows the merit of any research. The current format of this manuscript is very hard to understand for general readers like me.
We have revised the description and interpretation of the results to show the merits of our research. The current format of this manuscript allows its easy comprehension by general readers.
Another drawback is the arrangement of figures. It seems to me that the authors are very reluctant to rearrange their figures. The texts of several figures are very small and faint. If I can not read it then how come I comment? The author answered that the figure will be clearer if the picture will open in illustration software. However, they can easily increase the size of their texts for better viewing. I am very sorry to say that the current formats of their Figures are not up to the mark. In Figure 3A-3D, it is very hard to detect the color. The author can easily make it visible by adjusting brightness of the picture. Anyway, it is author’s choice. But the point is that they must need to provide improve figures to think forward.
We have adjusted the brightness of Figs 3A-3D and uploaded Figs 3A-3D and Figs 1E-1M separately. We have uploaded our figure files to the Preflight Analysis and Conversion Engine (PACE) digital diagnostic tool and ensured that the figures meet the PLOS requirements.
Data deposition is another major issue to me. The author was asked to deposit the metabolomics data to any open resource. They reply that they will deposit it. But when? After acceptance? They need to provide the accession number in this manuscript. However, the authors mentioned that all relevant data are within the manuscript, which is not true. This activity is against the PLoS guideline.

We have deposited the data in protocols.io to enhance the reproducibility of our results. The DOI link is [dx.doi.org/10.17504/protocols.io.bpyrmpv6](https://dx.doi.org/10.17504/protocols.io.bpyrmpv6).

Reviewer #3: (No Response)

7. PLOS authors have the option to publish the peer review history of their article ([what does this mean?](https://journals.plos.org/plosone/s/editorial-and-peer-review-process#loc-peer-review-history)). If published, this will include your full peer review and any attached files.


If you choose “no”, your identity will remain anonymous but your review may still be made public.


**Do you want your identity to be public for this peer review?** For information about this choice, including consent withdrawal, please see our [Privacy Policy](https://www.plos.org/privacy-policy).

Reviewer #2: No

Reviewer #3: No

[NOTE: If reviewer comments were submitted as an attachment file, they will be attached to this email and accessible via the submission site. Please log into your account, locate the manuscript record, and check for the action link "View Attachments". If this link does not appear, there are no attachment files.]

While revising your submission, please upload your figure files to the Preflight Analysis and Conversion Engine (PACE) digital diagnostic tool, <https://pacev2.apexcovantage.com/>. PACE helps ensure that figures meet PLOS requirements. To use PACE, you must first register as a user. Registration is free. Then, login and navigate to the UPLOAD tab, where you will find detailed instructions on how to use the tool. If you encounter any issues or have any questions when using PACE, please email PLOS at [figures@plos.org](mailto:figures@plos.org). Please note that Supporting Information files do not need this step.

We have uploaded our figure files to the Preflight Analysis and Conversion Engine (PACE) digital diagnostic tool and ensured that the figures meet the PLOS requirements.

*In compliance with data protection regulations, you may request that we remove your personal registration details at any time.* [*(Remove my information/details)*](https://www.editorialmanager.com/pone/login.asp?a=r)*. Please contact the publication office if you have any questions.*
