# Supplementary material for: Transplantation of chicken egg white extract-induced rabbit PBMCs as a treatment for renal ischemia-reperfusion injury in rabbits
Source: PLoS One. 2020 Dec 28;15(12):e0244160. doi: 10.1371/journal.pone.0244160 (PMC7769466; doi:10.1371/journal.pone.0244160)
Supplement: Attachment — Submitted filename: 7-Response to Reviewers.doc [file pone.0244160.s008.doc]

PONE-D-20-22977R4
Transplantation of Chicken Egg White Extract-Induced Rabbit PBMCs as a Treatment for Renal Ischemia-Reperfusion Injury in Rabbits
PLOS ONE

Dear Dr. Ruan,

Thank you for submitting your manuscript to PLOS ONE. After careful consideration, we feel that it has merit but does not fully meet PLOS ONE’s publication criteria as it currently stands. Therefore, we invite you to submit a revised version of the manuscript that addresses the points raised during the review process.

Specifically, please revise the subheadings of the results section according to the suggestions given below in the Additional Editor Comments (if provided) section.

We have revised the subheadings of the results section according to the suggestions given below in the Additional Editor Comments (if provided) section.

Please submit your revised manuscript by 11 December 2020. If you will need more time than this to complete your revisions, please reply to this message or contact the journal office at [plosone@plos.org](mailto:plosone@plos.org). When you're ready to submit your revision, log on to https://www.editorialmanager.com/pone/ and select the 'Submissions Needing Revision' folder to locate your manuscript file.

Please include the following items when submitting your revised manuscript:

- A rebuttal letter that responds to each point raised by the academic editor and reviewer(s). You should upload this letter as a separate file labeled 'Response to Reviewers'.
- A marked-up copy of your manuscript that highlights changes made to the original version. You should upload this as a separate file labeled 'Revised Manuscript with Track Changes'.
- An unmarked version of your revised paper without tracked changes. You should upload this as a separate file labeled 'Manuscript'.

If you would like to make changes to your financial disclosure, please include your updated statement in your cover letter. Guidelines for resubmitting your figure files are available below the reviewer comments at the end of this letter.

If applicable, we recommend that you deposit your laboratory protocols in protocols.io to enhance the reproducibility of your results. Protocols.io assigns your protocol its own identifier (DOI) so that it can be cited independently in the future. For instructions see: [http://journals.plos.org/plosone/s/submission-guidelines#loc-laboratory-protocols](http://journals.plos.org/plosone/s/submission-guidelines#loc-laboratory-protocols)

We have deposited our laboratory protocols in protocols.io to enhance the reproducibility of our results. The DOI link is [dx.doi.org/10.17504/protocols.io.bpyrmpv6](https://dx.doi.org/10.17504/protocols.io.bpyrmpv6).

We look forward to receiving your revised manuscript.

Kind regards,

Nazmul Haque
Academic Editor
PLOS ONE

Dear Editor,

Thank you very much for your letter and advice. We have revised the paper and would like to resubmit it for your consideration. We have addressed the reviewers’ comments, and the manuscript has been edited by American Journal Experts.

We hope that our revised manuscript is acceptable for publication and look forward to hearing from you at your earliest convenience.

Best wishes,

Guang-ping Ruan

Additional Editor Comments (if provided):

The authors have addressed almost all the issues. However, the following issues needed to be resolved before further consideration of this manuscript for publication.

1. Please revise the subheadings of the results section carefully. Few subheading are too long to consider as subheadings. Please revise the subheadings following the examples given below:

We have revised the subheadings of the results section carefully. We have revised the subheadings following the examples given below.

"TGF-β immunohistochemical analyses showed that the IOD of the induced cell groups was significantly reduced." change to "The IOD of the induced cell groups was significantly reduced:"

"Masson’s trichrome staining showed that fibrosis was improved in the induced group" change to "Fibrosis was improved in the induced group:"

"Renal PAS staining showed that the basement membrane did not display significant thickening in the induced group." change to "Thickening of the basement membrane was not observed in the induced group:"

"The results from renal tubular epithelial cell immunofluorescence showed that the transplanted cells were differentiated into tubular epithelial cells." change to "Transplanted cells differentiated into tubular epithelial cells:"

"The analysis of renal metabolomics pathways showed that the pyrimidine metabolism and phenylalanine, tyrosine and tryptophan biosynthesis pathways were significant." change to "Significant changes in the pyrimidine metabolism and phenylalanine, tyrosine and tryptophan biosynthesis pathways were observed"

[Note: HTML markup is below. Please do not edit.]


[NOTE: If reviewer comments were submitted as an attachment file, they will be attached to this email and accessible via the submission site. Please log into your account, locate the manuscript record, and check for the action link "View Attachments". If this link does not appear, there are no attachment files.]

While revising your submission, please upload your figure files to the Preflight Analysis and Conversion Engine (PACE) digital diagnostic tool, <https://pacev2.apexcovantage.com/>. PACE helps ensure that figures meet PLOS requirements. To use PACE, you must first register as a user. Registration is free. Then, login and navigate to the UPLOAD tab, where you will find detailed instructions on how to use the tool. If you encounter any issues or have any questions when using PACE, please email PLOS at [figures@plos.org](mailto:figures@plos.org). Please note that Supporting Information files do not need this step.

We have uploaded our figure files to the Preflight Analysis and Conversion Engine (PACE) digital diagnostic tool and ensured that the figures meet the PLOS requirements.

*In compliance with data protection regulations, you may request that we remove your personal registration details at any time.* [*(Remove my information/details)*](https://www.editorialmanager.com/pone/login.asp?a=r)*. Please contact the publication office if you have any questions.*
